# Cost-effectiveness of invitation to food supplementation early in pregnancy combined with multiple micronutrients on infant survival: analysis of data from MINIMat randomized trial, Bangladesh

**DOI:** 10.1186/s12884-015-0551-y

**Published:** 2015-05-28

**Authors:** Rubina Shaheen, Lars Åke Persson, Shakil Ahmed, Peter Kim Streatfield, Lars Lindholm

**Affiliations:** International Maternal and Child Health, Department of Women’s and Children’s Health, Akademiska sjukhuset, Uppsala University, Uppsala, SE 751 85 Sweden; Nossal Institute of Global Health, The University of Melbourne, Melbourne, Australia; icddr,b: International Centre for Diarrheal Disease Research, Bangladesh, 68 Shaheed Tajuddin Ahmed Sarani Mohakhali, Dhaka, 1212 Bangladesh; Department of Public Health and Clinical Medicine, Umeå University, Umeå, Sweden

**Keywords:** Cost-effectiveness, Prenatal food supplementation, Micronutrient supplementation, Infant mortality, Bangladesh

## Abstract

**Background:**

Absence of cost-effectiveness (CE) analyses limits the relevance of large-scale nutrition interventions in low-income countries. We analyzed if the effect of invitation to food supplementation early in pregnancy combined with multiple micronutrient supplements (MMS) on infant survival represented value for money compared to invitation to food supplementation at usual time in pregnancy combined with iron-folic acid.

**Methods:**

Outcome data, infant mortality (IM) rates, came from MINIMat trial (Maternal and Infant Nutrition Interventions, Matlab, ISRCTN16581394). In MINIMat, women were randomized to early (E around 9 weeks of pregnancy) or usual invitation (U around 20 weeks) to food supplementation and daily doses of 30 mg, or 60 mg iron with 400 μgm of folic acid, or MMS with 15 micronutrients including 30 mg iron and 400 μgm of folic acid. In MINIMat, EMMS significantly reduced IM compared to UFe60F (U plus 60 mg iron 400 μgm Folic acid). We present incremental CE ratios for incrementing UFe60F to EMMS. Costing data came mainly from a published study.

**Results:**

By incrementing UFe60F to EMMS, one extra IM could be averted at a cost of US$907 and US$797 for NGO run and government run CNCs, respectively, and at US$1024 for a hypothetical scenario of highest cost. These comparisons generated one extra life year (LY) saved at US$30, US$27, and US$34, respectively.

**Conclusions:**

Incrementing UFe60F to EMMS in pregnancy seems worthwhile from health economic and public health standpoints.

**Trial registration:**

Maternal and Infant Nutrition Interventions, Matlab; ISRCTN16581394; Date of registration: Feb 16, 2009.

## Background

The Bangladesh Integrated Nutrition Project (BINP) and its successor National Nutrition Program (NNP) implemented food supplementation programs for malnourished pregnant and lactating women and undernourished children less than two years of age [1, 2]. These programs aimed to reduce under-nutrition by improving maternal, fetal and childhood nutritional status, including birth weight (BW). In a previous paper we have shown positive effects of prenatal food supplement on BW among malnourished Bangladeshi women [3], which was similar to findings in other countries [4–6].

It has been debated whether to move from the routine iron-folic acid supplementation in pregnancy to multiple micronutrients (MMS) in low- and middle-income settings [7]. This motivated UNICEF/WHO/United Nations University to manufacture MMS for testing purposes under the assumption that populations in these countries are deficient in several micronutrients [7]. Effects of prenatal MMS on offspring mortality have varied, ranging from increased risk of mortality [8, 9], over no difference [10] to significant reductions in mortality [11]. A meta-analysis concluded that MMS supplementation in pregnancy does not result in reduction of stillbirths or neonatal deaths, compared to iron-folic acid supplementation [12].

These studies were conducted in populations often at risk of both macro- (food) and micronutrient deficiencies and MMS was not given in combination with food supplements. Prenatal food supplementation in the Gambia was associated with 46 % reduction in mortality during the first week of life [13]. A Cochrane systematic review based on 13 trials reported that a balanced protein energy supplementation during pregnancy improved fetal growth and reduced stillbirth and neonatal death [14].

In a randomized trial with the acronym MINIMat (Maternal and Infant Nutrition Interventions, Matlab) we have shown a substantial reduction in infant mortality (IM) with early invitation to prenatal food supplementation (E - around week 9 pregnancy) combined with MMS (EMMS) compared to invitation to food supplementation at usual time (U- around week 20) in pregnancy and 60 mg iron 400 μgm folic acid (UFe60F) [15].

Since there are questions on the affordability of large-scale food supplementation interventions and whether or not to move from the routine iron folic acid supplementation to MMS, we examined if EMMS represented value for money. We present incremental cost-effectiveness ratios (ICERs) for incrementing usual invitation to food supplementation combined with iron-folic acid to invitation to food supplementation early in pregnancy combined with multiple micronutrients for women in rural Bangladesh.

## Methods

### Study design and study population

Outcome data came from MINIMat trial (Maternal and Infant Nutrition Interventions, Matlab, Trial Registration: ISRCTN16581394). MINIMat was a randomized trial [15], conducted in Matlab, a sub-district in Chandpur district in Bangladesh. In Matlab, the International Centre for Diarrheal Disease Research, Bangladesh (icddr,b) operates a Health and Demographic Surveillance System (HDSS) since 1966. Details regarding design, randomization, treatment groups, and study profile of MINIMat study are published elsewhere [15]. In brief, MINIMat was a randomized factorial experiment, where pregnant women (n = 4436) irrespective of their nutritional status were randomized to early (E, at about 9 weeks of pregnancy), or usual invitation (U, at about 20 weeks of pregnancy) to daily (6 days a week) food supplement and to one of the three types of micronutrient capsules, 30 mg iron and 400 μgm folic acid, or 60 mg iron and 400 μgm folic acid, or multiple micronutrients (MMS) with 15 micronutrients including 30 mg iron and 400 μgm folic acid. The food supplementation provided about 608 kcal (made of roasted rice powder 80 g, roasted pulse powder 40 g, soybean oil 6 g and molasses 20 g). MMS contained recommended daily allowances of 13 micronutrients and 30 mg iron 400 μgm folic acid [7]. This resulted in six intervention groups, EFe30F (n = 739), EFe60F (n = 738), EMMS (n = 740), UFe30F (n = 741), UFe60F (n = 738), and UMMS (n = 740). Randomization of invitation to food supplement was not blinded but that of micronutrient supplementation was double-masked. This study was approved by the icddr,b ethical review committee. Informed written consent was obtained from all participants.

### Outcome data and alternatives

Outcome data, IM, came from intent-to-treat analysis of MINIMat trial comparing UFe60F and EMMS arms, HR 0.38 (95 % CI: 0.18 to 0.78) [15]. Therefore, we compared the alternatives UFe60F, and EMMS in MINIMat.

Adherence to food and micronutrients also came from MINIMat study; 60 food packets and 113 capsules in UFe60F arm, and 94 food packets and 107 capsules in EMMS arm [16].

We assumed the effects of MINIMat intervention were accumulated from June 2002 through June 2004 and this can be represented by reductions in IM in the EMMS arm compared to the UFe60F arm.

By using life expectancy (LE) at birth, 70 years in the year 2012 [17], we calculated the average LY that can be saved by avoiding one IM; this was 29.99 years when discounted at 3 % and 20.31 years when discounted at 5 %. Since we adjusted all costs using consumer price index, to remain consistent we also adjusted the health gains by discounting the LYs gained; this resulted in treating this nutrition intervention similarly as with other sectors of the economy [18].

### Time frame

MINIMat intervention was distributed from September 2001 to end of 2003. The costing study was conducted from October 1999 to March 2000 [19]. In 2002, we retrieved additional cost data from BRAC, a non-governmental organization (NGO) responsible for implementation of food supplementation. In 2003 we also collected the cost data for micronutrients and some staff salaries from MINIMat administration at icddr,b. We believe the cost data collected by Khan and Ahmed from October 1999 to March 2000 [19] and the additional cost data collected by us in 2002 and 2003 reasonably represented the same period as the outcome data.

### Cost data

The direct cost for the intervention included food and micronutrient supplements, staff, training and meeting, administration, capital, community volunteer time, and recurrent activities. The indirect cost included the cost of participants’ time.

Most cost data were available from Khan and Ahmed [19], while data on cost of micronutrients and some staff costs were obtained from the MINIMat project administration. Figures for all cost items from Khan and Ahmed [19] were converted to Bangladeshi Taka (BDT) 79.3823 per US$1, the average exchange rate for 2013 [20]. Khan and Ahmed reported costs for NGO run and government run community nutrition centres (CNCs). We presented all costs for these delivery modes as well as under a hypothetical highest cost scenario combining the highest cost for each item presented for NGO run and government run CNCs. For the last, for example, for food cost we took the figure for NGO run CNCs but for staff cost we took the figure for government run CNCs.

CNCs operated for malnourished pregnant and lactating women and children under two years of age. Per day at the NGO run CNCs, there were 9.36 pregnant women, 9.71 lactating women and, and 10.29 under two-year-olds. In total 19.07 pregnant and lactating women represented 19.07 adult equivalents and, 10.29 children represented 5.15 adult equivalents equal to 24.22 adult equivalents. This was because each pregnant or lactating woman was offered four packets and each child was offered two packets of food supplement; thus, two children equals to one adult. There were 7579 (24.22*313) person days per year (313 working days per year; CNCs were closed on Fridays). Pregnant women represented 2930 (9.36*313) person days a year and used about 32 % (9.36/29.36*100) of working time at the CNC [19]. We assumed pregnant women received comparable services as children did, which we believe was a conservative assumption for CE analysis. Therefore, for calculation of food cost we used adult equivalents and for other costs we used persons contributed by pregnant women. In total, 37.44 packets (9.36*4) were utilized per CNC per day by pregnant women, which were 39 % of total 97 (9.36*4 + 9.71*4 + 10.29*2) food packets. For all items, we calculated cost per CNC per year and then cost per pregnant woman per day. Food cost was multiplied by 0.39 (fraction of packets used) and, then, divided by 2930 (person days) to obtain costs per pregnant woman per day. For other items, costs per CNC per year were multiplied by 0.32 (proportion of working time) to represent pregnant women and, then, divided by 2930 to obtain cost per pregnant woman per day. In government run CNCs 6.33 pregnant women, 5.29 lactating women, and 4.38 children were enrolled resulting in 16 persons, 13.81 adult equivalents, and 55.24 food packets consumed. In this situation, pregnant women consumed 46 % food packets [(6.33*4)/55.24*100], and used 40 % time [6.33/16*100]. For the hypothetical highest cost scenario we used proportions of food consumed and time used in government run CNCs since this generated the highest cost figures.

Staff cost was from Khan and Ahmed [19], who derived that from the current local salary and benefits of BINP employees, and evaluated volunteers’ time using the salary level of similar workers in rural areas. Staff costs included salaries for the manager (BDT 10,000 per month), Community Nutrition Organizers, Community Nutrition Promoters, and helpers. The manager’s (responsible for NNP-related activities at sub-district level) salary was retrieved from the cost report of icddr,b. Increase in staff’s salaries over time was accounted for by a 40 % increase in staff salary from 2002. Training and meeting costs at sub-district level for 2002 were obtained from BRAC, who also provided administrative cost for 2000 to 2003, which was averaged: these costs at CNC levels were available from Khan and Ahmed [19]. We ignored administrative costs at BINP/NNP office.

Capital costs, space for CNCs, and instruments for screening, maternal height and weight measuring scales, were available from Khan and Ahmed [19]. From this cost we deducted cost for measuring scales (Salter scale and bathroom scale for measuring children’s and women’s weight, respectively) since in MINIMat all women were offered to participate in food supplementation intervention irrespective of their anthropometry. Khan and Ahmed calculated the salary of the Community Volunteers (women for preparing and serving food supplement) as community-donated time at the wage of helpers [19], which we considered appropriate: recurrent costs at the CNC that represented cost related to the goods procured locally and from outside the local area were available from Khan and Ahmed [19]. We ignored these costs for the sub-district, and central level in Dhaka.

Participant cost was estimated at cost of a laborer when labor cost is the lowest [21]. UNICEF supplied micronutrient capsules for trial purposes; the price was not subsidized. Assuming economic life of inputs, Khan and Ahmed [19] annualized all capital costs at 5 % discount rate, evaluated donated materials and resources using the market price of similar resources in the local area. We did not do any further discounting but adjusted all costs to the price levels for 2013 using consumer price index [17, 22]. All costs are presented in Table 1.

**Table 1:** Cost of food supplementation for pregnant women in Bangladesh by implementation strategy, NGO run community nutrition centres (CNSs), government (Govt.) run CNCs, and by a hypothetical highest cost scenario^a^

Cost items	NGO run CNC	Govt. run CNC	Highest cost
Food	25.30 (0.3187) [46.02]	15.70 (0.1978) [32.70]	30.03 (0.3783) [48.07]
Staff	3.09 (0.0389) [5.62]	4.78 (0.0602) [9.95]	4.78 (0.0602) [7.65]
Training and meeting	0.91 (0.0114) [1.65]	1.09 (0.0138) [2.28]	1.13 (0.0142) [1.80]
Administration	0.64 (0.0081) [1.17]	0.80 (0.0101) [1.66]	0.80 (0.0101) [1.28]
Capital (space)	1.17 (0.0147) [2.12]	1.35 (0.0170) [2.81]	1.45 (0.0182) [2.31]
Community Volunteers’ time	0.65 (0.0082) [1.18]	0.81 (0.0101) [1.68]	0.81 (0.0101) [1.29]
Recurrent	0.37 (0.0047) [0.67]	0.64 (0.0081) [1.33]	0.64 (0.0081) [1.03]
Participant time	22.85 (0.2878) [41.57]	22.85 (0.2878) [47.60]	22.85 (0.2878) [36.58]
Cost/pregnant woman/day for food	54.98 (0.6926) [100 %]	48.01 (0.6048) [100 %]	62.47 (0.7869) [100 %]

^a^ 1 US$ =79.3823 Bangladeshi Taka (BDT) at exchange rate for the year 2013 (Reference number 20). Costs represent per pregnant woman per day in BDT (US$) [% total cost]. Cost per capsule for iron folic acid is BDT 0.457 (US$ 0.00575), and multiple micronutrients is BDT 1.371 (US$ 0.017272). Calculations are presented for NGO run CNCs, government run CNCs, and under a hypothetical highest cost scenario combining costs from the first two options. Sources are reference numbers 15, 17, 19, and 21

### Analyses

For ICERs for extra IM averted we calculated the difference of costs for supplementing 1000 pregnant women in EMMS and UFe60F arms, difference of IM rates between these two arms and then divided the cost difference by the difference of IM rates. For ICERs for LY saved, the same steps were followed after multiplying the IM averted (IM rate in UFe60F – IM rate in EMMS) with discounted figures of LE at birth. For sensitivity analyses, we used the lower and upper limits of 95 % CI of HR from the intent-to-treat analyses comparing EMMS and UFe60F arms in MINIMat, and converted the resulted number of infant deaths to IM rates. The above-described steps for calculating ICERs were then followed and ICERs for extra IM averted and extra LY gained were calculated.

## Results

Using the adherence levels and costs from Table 1, we calculated, by using delivery modes NGO run CNC, government run CNC, and a hypothetical highest cost scenario supplementing 1000 pregnant women would cost US$42,207, US$36,939, and US$47,865 respectively for UFe60F. These figures were US$66,953, US$58,699, and US$75,817 for EMMS (Table 2).

**Table 2:** Infant mortality (IM) rates per 1000 live births, adherence to intervention and cost for supplementing each and 1000 pregnant women for NGO run community nutrition centres (CNSs), government (Govt.) run CNCs and a hypothetical highest cost scenario^a^

Alternative	IM rate	Adherence to food packets	Adherence to micronutrient capsules	Cost for one woman (US$)			Cost for 1000 women (US$)		
				NGO run CNC	Govt. run CNC	Highest cost	NGO run CNC	Govt. run CNC	Highest cost
UFe60F^b^	44.1	60	113	42.207	36.939	47.865	42,207	36,939	47,865
EMMS^c^	16.8	94	107	66.953	58.699	75.817	66,953	58,699	75,817

^a^UFe60F = invitation to prenatal food supplementation at usual time in pregnancy (at about week 20) and 60 mg iron 400 μgm folic acid. EMMS = early invitation to prenatal food supplement (at about 9 week gestation) plus multiple micronutrients (MMS); Cost per pregnant woman per day for food US$0.6926, US$0.6048, and US$0.7869 for NGO run CNCs, government run CNCs and a hypothetical highest cost scenario, MMS US$0.017272, iron-folic acid US$0.0057574. Sources for IM rates reference number 15, and adherence to the intervention reference number 16

^b^cost of supplementing 1000 pregnant women with UFe60F for NGO run CNCs, government run CNCs, and for a hypothetical highest cost scenario were, US$42,207 [(0.6926*60*1000) + (0.005757*113)*1000)], US$36,939 [(0.6048*60*1000) + (0.005757*113)*1000)], and US$47,865 [(0.7869*60*1000) + (0.005757*113)*1000)], respectively

^c^cost of supplementing 1000 pregnant women with EMMS for NGO run CNCs, government run CNCs, and for a hypothetical highest cost scenario were, US$66,953 [(0.6926*94*1000) + (0.017272*107*1000)], US$58,699 [(0.6048*94*1000) + (0.017272*107*1000)], and US$75,817 [(0.7869*94*1000) + (0.017272*107*1000)], respectively

### Incremental cost-effectiveness ratios

The ICERs for one extra IM averted by switching from UFe60F to EMMS were US$907, and US$797, respectively, for NGO run and government run CNCs (Table 3). This figure for the hypothetical highest cost scenario would be US$1024 for one extra IM averted (Table 3). With 3 % discount rate of LE at birth corresponding figures for LY saved for switching from UFe60F to EMMS ranged from US$27 to US$34, at the level of costs by the above two service delivery modes and by the highest cost scenario (Table 3). The corresponding LY gained figures for LE discounted at 5 % rate were, US$45, US$39, and US$51, respectively.

**Table 3:** Incremental cost-effectiveness ratios (ICERs) for cost per extra infant mortality (IM) averted and extra life year (LY) saved: for NGO run community nutrition centres (CNSs), government (Govt.) run CNCs and for a hypothetical highest cost scenario^a^

Switching alternatives	Incremental IM averted	Incremental Costs (US$)			ICERs for one extra unit of gain (US$)								
					IM averted			LY saved^b^			LY saved^c^		
		NGO run CNC	Govt. run CNC	Highest cost	NGO run CNC	Govt. run CNC	Highest cost	NGO run CNC	Govt. run CNC	Highest cost	NGO run CNC	Govt. run CNC	Highest cost
UFe60F to EMMS^d^	27.3 (44.1 − 16.8)	24,746 (66,953 − 42,207)	21,760 (58,699 − 36,939)	27,952 (75,817 − 47,865)	907 (24,746/27.3)	797 (21,760/27.3)	1024 (27,952/27.3)	30 (24,746/819)	27 (21,760/819)	34 (27,952/819)	45 (24,746/554)	39 (21,760/554)	51 (27,952/554)
UFe60F to EMMS^e^													
Lower limit	36.14 (44.1 − 7.96)	24,746 (66,953 − 42,207)	21,760 (58,699 − 36,939)	27,952 (75,817 − 47,865)	685 (24,746/36.14)	602 (21,760/36.14)	774 (27,952/36.14)	23 (24,746/1084)	20 (21,760/1084)	26 (27,952/1084)	34 (24,746/734)	30 (21,760/734)	38 (27,952/734)
Upper limit	9.60 (44.1 − 34.50)				2577 (24,746/9.60)	2266 (21,760/9.60)	2911 (27,952/9.60)	85 (24,746/288)	76 (21,760/288)	97 (27,952/288)	126 (24,746/195)	112 (21,760/195)	143 (27,952/195)

^a^ UFe60F = invitation to prenatal food at usual time in pregnancy (at about 20 week) plus 60 mg iron 400 μgm folic acid, EMMS = early invitation to prenatal food supplement (at about 9 week) plus multiple micronutrients (MMS); IM rates per 1000 live births in UFe60F 44.1, and EMMS 16.8 [reference number 15]. LY saved = IM averted* life expectancy (LE) at birth [70 years in 2012, reference number 17] discounted at 3 % and 5 %, present value 29.99 and 20.31 years, respectively. Because of rounding, some estimates are same

^b^ Based on LY saved when LE at birth discounted at 3 %, present value 29.99 years; moving from UFe60F to EMMS, 27.3*29.99 = 819 years

^c^ Based on LY saved when LE at birth discounted at 5 %, present value 20.31 years; moving from UFe60F to EMMS, 27.3*20.31 = 554 years

^d^ ICER for moving from UFe60F to EMMs using the point estimate HR, 0.38 (95 % CI: 0.18 to 0.78)

^e^ ICERs for moving from UFe60F to EMMs using the lower and upper limits of 95 % CI of HR. The lower limit of 95 % CI of HR (0.18) is related to 4.74 infant deaths [Total deaths 10 out of 595 live births; 10/0.38*0.18 = 4.74] resulting in IM rate of 7.96/1000 live births (4.74/595*1000 = 7.96); the upper limit (0.78) is related to 20.53 deaths [10/0.38*0.78 = 20.53] resulting in IM rate 34.50/1000 live births (20.53/595*1000 = 34.50). Death rates in arms EMMS, 16.8/per 1000 live births and UFe60F, 44.10/1000 live births (reference number 15). LYs saved from lower limit of HR at 3 % live expectancy at birth, 36.14*29.99 = 1084, and at 5 % discount rate of life expectancy at birth, 36.14*20.31 = 734. These figures using upper limit of HR were, 9.60*29.99 = 288, and 9.60*20.31 = 195, respectively

### Sensitivity analyses

The IM rates associated with the lower limit of 95 % CI of HR from the intent-to-treat analysis showed ICERs for one extra IM averted by switching UFE60F to EMMS were US$685, and US$602, respectively for NGO run and government run CNCs, and US$774 for the hypothetical highest cost scenario (Table 3). ICERs related to LY saved with 3 % discount rate of LE at birth ranged from US$20 to US$26 for the above three options, and with 5 % discount rate of LE at birth ranged from US$30 to US$38 (Table 3).

The IM rates associated with the upper limit of 95 % CI of HR showed ICERs for one extra IM averted were US$2577, and US$2266, respectively for NGO run and government run CNCs; and US$2911 for the hypothetical highest cost scenario (Table 3). Under this circumstance, the ICERs for LY saved ranged from US$76 to US$97 at 3 % discount rate and US$112 to US$143 at 5 % discount rate of LE at birth (Table 3).

## Discussion

Our incremental CE analyses have shown by using actual delivery modes one extra IM could be averted at a cost of about US$797 to US$907 by switching from UFe60F to EMMS in pregnancy. Our analyses have also shown one extra LY can be saved at a cost of US$27 to US$30 by switching from UFe60F to EMMS. We consider the amount of expenditures to attain these gains, which is less than per capita gross domestic product (GDP) US$958 in the year 2013 in Bangladesh [17] represents good value for money.

### Validity of data

The effect data in the CE analysis are considered to be valid, as the trial design offered unbiased estimates of treatment effects. All pregnant women in the area were invited to participate in the study, and the pool of pregnant women under trial was probably representative of rural settings in Bangladesh, where women in general are short, and there is occasional food insecurity. Therefore the ICERs presented may be considered relevant for similar settings. The cost data were collected within the time frame and situation similar to that of the MINIMat trial and we believe it reasonably represents costs incurred by these interventions.

### Explaining the results and comparison with other studies

As the overall cost of these programs is high, an evaluation by Hossain *et al.* questioned the affordability and cost-effectiveness of the program [23]. By design that ecological study compared childhood nutritional status in BINP and non-BINP areas and did neither provide information on baseline nutritional status, nor did it control for confounding factors. Further, pregnant women were ignored when investment for them is likely to incur positive effects over generations. Thus, we consider the evaluation by Hossain *et al.* questioning the US$60 million budget as erroneous and incomplete.

In a micronutrient trial [8] conducted in the neighboring country Nepal, the study participants were rural pregnant women without any inclusion criteria related to their nutritional status and the authors indicated increased risk of perinatal mortality associated with prenatal MMS supplementation compared to iron-folic acid [8]. In a cluster-randomized trial in Indonesia, prenatal MMS supplementation compared to iron-folic acid was associated with 18 % reduction of IM [11]. In MINIMat, offspring mortality decreased for infants in EMMS arm compared to UFe60F arm [15] and larger reductions occurred for that intervention among women having lower education [16]. The difference between the above trials and MINIMat is that invitation to food supplementation early in pregnancy is combined with the multiple micronutrients, and the reduction in mortality in MINIMat was found as a combined effect of early food and MMS. Our results indicate the better survival among infants is the effects of increased food intake from around week 10 in pregnancy plus the addition of 15 micronutrients from around week 14 on fetal health.

The cost per extra IM averted estimates, US$757 to US$907, from government run and NGO run CNCs are less than per capita GDP, US$958, in Bangladesh in the year 2013 [17], which are within the threshold for relative cost-effectiveness of an intervention suggested by the Commission of Macroeconomics and Health [24]. The Commission sets threshold value as less than GDP highly cost effective and 1 to 3 times GDP cost effective [24]. Our costs per extra LY saved estimates are far below the per capita GDP.

A community based newborn care intervention in Bangladesh has shown that a neonatal death could be averted at a cost of US$ 2939 [25], which is much higher than our estimates. Further, our cost per one extra IM averted figures is lower than that reported by the World Bank in an evaluation of BINP: US$2300 (lower estimate) and US$4100 (upper estimate) as the cost for death averted [26], and is within the range suggested for packages of neonatal health interventions (US$ 17–3100: average 2100/death averted) [27]. Further, our estimates US$27 to USS30 per LY saved for switching from UFe60F to EMMS are lower than US$211 per LY saved from participatory women’s group intervention in Nepal [28]. The cost of implementation could possibly be reduced through combining the increment into a package with other services for pregnant women. But this is likely to increase workload, and would require increased capacity in the perinatal services provided by primary health centres and would entail additional monitoring.

A recent study indicated that monitoring and start-up costs might not be trivial [29]. The costing study by Khan and Ahmed has shown though higher percentage of target population was enrolled in NGO run CNCs, actual show up of enrollees was higher in government run CNCs, 60 % vs., 78 % [19]. This implies a reasonable participation and effect can be expected if interventions are delivered through government run CNCs where monitoring is an inbuilt mechanism for all perinatal services. In the costing study, to a large extent, the start-up costs were covered both when delivered through NGO and government run CNCs.

#### Cost and effect variation, and implications for resultant ICERs

We presented cost per pregnant woman for NGO run and government run CNCs, and for a hypothetical highest cost scenario. Since food cost comprises the largest component cost, low food cost if delivered through government run CNCs resulted in substantially lower ICERs for extra IM averted compared to ICERs for NGO run CNCs and the hypothetical highest cost scenario. These differences are also evident in case of one extra IM averted based on effect estimates from the upper and lower limits of 95 % CI of HR comparing UFe60F and EMMS. Depending on prevalence of risk factors for IM and responsiveness of these risk factors to prenatal food and micronutrient interventions, the effect estimates as captured in 95 % CI of HR may result in substantial reductions in IM from prenatal food and micronutrient interventions and highly favorable ICERs may be observed. Since actual show-ups of enrollees were higher for government run CNCs [19] possibly due to the availability of other maternal and child health services, the ICERs we presented indicate favorable outcomes with reasonable costs may be expected by scaling up prenatal food and micronutrient interventions in government health care system.

#### Limitations

We do not have data on treatment cost for the side effects of micronutrients and other intangible costs. Similarly, our analysis is based on one positive outcome, IM reduction; we did not include other tangible and intangible benefits of the intervention that may have occurred [30].

## Conclusions

Our analysis shows that at US$797 to US$907 and US$27 to US$30 respectively, one extra IM can be averted and one extra LY can be saved by switching from invitation to food supplementation at usual time in pregnancy and iron-folic acid supplementation to an early initiation to food supplementation combined with MMS. These figures are below per capita GDP, US$958, for the year 2013 in Bangladesh. Compared to the economic venture, the health gains can be considered high in low-income countries and in the global context. These results should aid decision-making that prioritizes pregnant women’s health. Further research is needed to understand if better infant survival can be achieved by improving diet during early pregnancy.

## Abbreviations

CE: Cost-effectiveness
MMS: Multiple micronutrient supplements
IM: Infant mortality
MINIMat: Maternal and infant nutrition interventions, Matlab
EMMS: Invitation to food supplementation early in pregnancy and multiple micronutrients
UFe60F: Invitation to food supplementation at usual time in pregnancy and 60 mg iron plus 400 μgm folic acid
EFe30F: Invitation to food supplementation early in pregnancy and 30 mg iron plus 400 μgm folic acid
EFe60F: Invitation to food supplementation early in pregnancy and 60 mg iron plus 400 μgm folic acid
UFe30F: Invitation to food supplementation at usual time in pregnancy and 30 mg iron plus 400 μgm folic acid
UMMS: Invitation to food supplementation at usual time in pregnancy plus multiple micronutrients
LY: Life year
BINP: Bangladesh integrated nutrition project
NNP: National nutrition program
BW: Birth weight
ICERs: Incremental cost-effectiveness ratios
icddr,b: International centre for diarrheal disease research, Bangladesh
HDSS: Health and demographic surveillance system
LE: Life expectancy
BRAC: Bangladesh rural advancement committee
NGO: Non-governmental organization
BDT: Bangladeshi taka
CNCs: Community nutrition centres
GDP: Gross domestic product
